# Risk of Recall Associated With Modifications to High-risk Medical Devices Approved Through US Food and Drug Administration Supplements

**DOI:** 10.1001/jamanetworkopen.2023.7699

**Published:** 2023-04-12

**Authors:** Jonathan R. Dubin, Jonathan R. Enriquez, An-Lin Cheng, Hunter Campbell, Akin Cil

**Affiliations:** 1 Department of Orthopedics, University of Missouri–Kansas City; 2 University Health Medical Center, Kansas City, Missouri; 3 Missouri State Orthopedics Association, Jefferson City, Missouri; 4 Cardiology Fellowship Training Program, University of Texas Southwestern Medical Center, Dallas; 5 Saint Luke’s Mid America Heart Institute, Kansas City, Missouri; 6 Research and Statistical Consult Service, University of Missouri–Kansas City; 7 Department of Biomedical and Health Informatics, University of Missouri–Kansas City; 8 currently a medical student at University of Missouri–Kansas City School of Medicine; 9 Department of Orthopedics, University of Missouri–Kansas City; 10 Department of Orthopedics, University Health Medical Center, Kansas City, Missouri

## Abstract

**Importance:**

High-risk medical devices approved by the US Food and Drug Administration (FDA) can undergo modifications to their original premarket approval (PMA) via 1 of 5 types of supplements. Only panel track supplements (approximately 1%) require clinical data for approval. The association between device modifications and risk to patient safety has not previously been analyzed.

**Objective:**

To determine the association between PMA supplements and the risk of any device recall and high-risk (class 1) recall.

**Design, Setting, and Participants:**

In this cohort study, the FDA database was queried for original devices approved via PMA from January 1, 2008, through December 31, 2019. Supplement and recall data were obtained for these devices from January 1, 2008, through December 31, 2021, giving a minimum 2-year follow-up after initial approval. Data were analyzed from July 6 to August 6, 2022. Retrospective, time-to-event analysis investigated the association between the number and type of supplements and risk of recall.

**Exposures:**

Supplements submitted by manufacturers for FDA approval to modify devices.

**Main Outcomes and Measures:**

A mixed-effects Cox proportional hazards regression model with frailty terms was used, modeling device recall as an outcome variable during the observation period. A second model was performed for class 1 (high-risk) recall. Explanatory variables are the number of supplements, number of panel track supplements, and cardiovascular devices. Multivariable analysis was performed to identify independent risk factors for recall with hazard ratios (HRs) as the main end point.

**Results:**

A total of 373 original PMA devices with 10 776 associated supplements were included in the analysis. A median 2.5 (IQR, 1.2-5.0) supplements per device were approved annually. Cardiovascular devices contributed 138 supplements (37.0%), followed by microbiology with 45 (12.1%). No other specialty contributed more than 10%. Multivariable analysis demonstrated that each increase of 1 supplement per year was associated with increased risk of recall (HR, 1.28 [95% CI, 1.15-1.44]; *P* < .001). For class 1 recall, increased number of supplements (HR, 1.32 [95% CI, 1.06-1.64]; *P* = .01) and cardiovascular vsnoncardiovascular classification of devices (HR, 3.51 [95% CI, 1.15-10.72]; *P* = .03) were significantly associated with an increased risk of recall.

**Conclusions and Relevance:**

The findings of this cohort study suggest that PMA supplements are associated with an approximately 30% increased risk of any recall and class 1 recall. The FDA processes for approving modifications to high-risk medical devices should be reevaluated to optimize patient safety and public health.

## Key Points

**Question:**

Are modifications to high-risk medical devices approved through US Food and Drug Administration supplements associated with an increased risk of any recall and high-risk (class 1) recall?

**Findings:**

In this cohort study with a time-to-event analysis of 373 devices with premarket approval between 2008 and 2019 and 10 766 subsequent supplements, devices with a greater number of supplements, greater number of panel track supplements, and cardiovascular vs noncardiovascular classification were associated with device recalls.

**Meaning:**

These findings suggest that increased oversight of the US Food and Drug Administration supplement approval process and improvements to postmarketing surveillance systems should be considered to mitigate risks to patient safety.

## Teaser

This cohort study identifies whether US Food and Drug Administration approval of modifications to high-risk medical devices via supplements is associated with an increased risk of any recall and class 1 recall.

## Introduction

High-risk medical devices, or class III devices, “sustain or support life, are implanted, or present potential unreasonable risk of illness or injury.”^1^ These devices, such as cardiac pacemakers or defibrillators, breast and cochlear implants, and other implanted prosthetic devices, constitute approximately 1% of the nearly 3000 devices authorized annually by the US Food and Drug Administration (FDA).^2,3^ In recognition of the inherent risks posed by these devices, the FDA imposes stricter premarket review standards compared with those requested of lower-risk devices, including necessitating clinical trials in a process called premarket approval (PMA).^4,5^ Despite these safeguards, devices approved via PMA harbor 3 times the risk of any recall and 7 times the risk of a class 1 (highest risk) recall when compared with those devices cleared through the less stringent 510(k) pathway used for nearly all moderate-risk devices (class II).^4^ Little published data exist elucidating reasons for this, and 1 possibility is the extensive alterations manufacturers make to already approved devices.

To facilitate incremental improvements in safety and effectiveness, the FDA allows manufacturers to modify previously approved devices by submitting supplements to the original PMA application. Premarket approval supplements have been described previously.^6,7^ Briefly, there are 5 supplement types, each purposed with handling modifications of various perceived risks. Panel track supplements (PTS) pose the greatest risk and typically handle expanding indications and some major design changes. Used least frequently, they are the only supplements to require clinical data for support. Other supplement types include 180-day (major design changes, labeling changes), real-time (minor design changes), special (labeling designed to improve safety), and 30-day notice or 135-day supplement (manufacturing changes [eg, sterilization, cleansing, or welding of parts]).^6,7^

Estimates for the median number of supplements per original PMA range from 6.5 to over 50.^7,8^ Previous investigators have expressed concerns that modified devices may bear little resemblance to the device originally approved.^7,8,9^ Two brands of cardiac defibrillator leads (Sprint Fidelis [Medtronic] and Riata [St Jude Medical]) were recalled after being approved by supplements due to high rates of lead failures.^7^ Similar regulatory failures have been reported in otolaryngology, with a cochlear implant (CII [Advanced Bionics]) being recalled due to otogenic meningitis linked to an electrode positioner approved via supplements,^9^ along with additional examples in orthopedics.^8^

To date, no prior studies have examined the association between PMA supplements and patient safety. The primary purpose of this investigation is to identify whether PMA supplements are associated with an increased risk of any recall and class 1 recall. Secondary end points include analysis of any association of PTS and cardiovascular device with recall, considering their higher perceived safety risks. The final aim was to describe reasons for the recall of PMA devices.

## Methods

### Device Study-Group Identification

This cohort study followed the Strengthening the Reporting of Observational Studies in Epidemiology (STROBE) reporting guideline. The study was exempted from institutional review board approval by the University of Missouri–Kansas City owing to the use of publicly available data. From the FDA website, the pma.zip file was downloaded in January 2022 and extracted into an Excel spreadsheet, version 16.0.1 (Microsoft Corporation).^10^ The file contains information on all original PMAs and supplements, including approval dates, supplement types, supplement reasons, and advisory committees. Advisory committees are specialty specific and are used by the FDA to inform decision makers on medical recommendations regarding devices. We used FDA-determined advisory committee assignments to classify each device into fields for analysis. Filtering isolated those devices with an original PMA approval between January 1, 2008, and December 31, 2019, yielding 373 unique devices. Next, supplements were filtered to include those submitted as modifications to 1 of these 373 original PMA devices, and were approved by December 31, 2021, to allow a minimum of 2 years for each device to accrue modifications. This yielded 10 776 associated supplements. The 135-day supplements were combined with 30-day notice, as the former is issued when the FDA requires additional time to make a determination regarding a 30-day notice.^11^

### Recall Data Extraction

Recall data were obtained from the FDA’s public PMA database.^12^ The unique PMA number for each of the 373 original devices included in the study was downloaded on this database, and recall information, including the FDA-determined root cause, class, and date of recall was obtained and recorded. The FDA lists 44 unique root causes for recall on its database that they assign after their analysis of the issue. While the database provides a brief (typically 1 sentence) explanation of the recall from the manufacturer, we used *root causes* in our descriptive analyses to minimize subjectivity.

Because multiple parts of the same device can be recalled, recalls with the same date were counted only once. Recall data were abstracted in January 2022, with a study end date of December 31, 2021, to allow for a minimum of 2 years of follow-up for recalls to occur.

The FDA codifies recalls into 3 tiers based on perceived risk: class 1 is highest risk, class 2 is moderate risk, and class 3 is lowest risk. Specific definitions have been described previously.^13^

### Statistical Analysis

Data were analyzed from July 6 to August 6, 2022. A mixed-effects analysis with Cox proportional hazards regression model using frailty terms was conducted to model device recall as an outcome variable during the observation period. The frailty model was used because it is possible that each device had repeated recalls throughout the years. A second model was used for class 1 recall. Explanatory variables include the number of supplements, number of PTS, and cardiovascular device (yes or no). Cumulative supplements were calculated throughout the year and treated as independent variables. Significance levels were set at *P* < .05, and tests were 2 sided. Analyses were performed using RStudio, version 1.4.1717 (R Project for Statistical Computing), for 2009 to 2021 and the 2022 survival package from Therneau.^14^

Testing of the proportional hazards assumption was conducted with proportional hazard hypothesis testing. The results showed cumulative supplements violated this assumption; therefore, squared root transformation of cumulative supplements was calculated and used in the modeling.

## Results

From 2008 to 2019, 373 original devices were approved via PMA, with a slight increasing trend over time. The largest specialty consisted of cardiovascular devices, with 138 (37.0%), followed by microbiology with 45 (12.1%). No other specialty contributed 10% or more of the total (Table 1).

**Table 1.** Devices and Supplements by Specialty

Specialty	Original devices, No. (%)	Supplement type, No. (%)						Median (IQR) [maximum]	Mean (SD)
		Panel track	180-d	Real-time	Special	30-d Notice or 135-d	All		
Total No.	373 (100)	132 (100)	1801 (100)	1239 (100)	354 (100)	7250 (100)	10 776 (100)	NA	NA
Anesthesia	7 (1.9)	2 (1.5)	63 (3.5)	20 (1.6)	4 (1.1)	102 (1.4)	191 (1.8)	18 (8.5-40) [71]	27.3 (24.7)
Cardiovascular	138 (37.0)	63 (47.7)	840 (46.6)	578 (46.7)	167 (47.2)	4180 (57.7)	5828 (54.1)	24.5 (10-59.3) [307]	42.2 (47.6)
Clinical chemistry	15 (4.0)	14 (10.6)	60 (3.3)	110 (8.9)	25 (7.1)	525 (7.2)	734 (6.8)	38 (29.5-70) [132]	48.9 (33.3)
Ear, nose, and throat	4 (1.1)	2 (1.5)	34 (1.9)	22 (1.8)	6 (1.7)	66 (0.9)	130 (1.2)	40.5 (27.8-45.3) [46]	32.5 (17.5)
Gastroenterology and urology	15 (4.0)	1 (0.8)	78 (4.3)	56 (4.5)	27 (7.6)	332 (4.6)	494 (4.6)	19 (9-32) [231]	32.9 (54.4)
General and plastic surgery	19 (5.1)	9 (6.8)	69 (3.8)	38 (3.1)	19 (5.4)	207 (2.9)	342 (3.2)	11 (4-28) [58]	18.0 (15.2)
General hospital	3 (0.8)	0	22 (1.2)	25 (2.0)	9 (2.5)	74 (1.0)	130 (1.2)	57 (29.0-64.5) [72]	43.3 (30.6)
Immunology	4 (1.1)	0	4 (0.2)	16 (1.3)	4 (1.1)	23 (0.3)	47 (0.4)	10.5 (1.5-20.8) [26]	11.8 (11.1)
Microbiology	45 (12.1)	3 (2.3)	134 (7.4)	121 (9.8)	28 (7.9)	744 (10.3)	1030 (9.6)	18 (13-27) [83]	22.9 (17.7)
Molecular genetics	5 (1.3)	0	1 (0.1)	1 (0.1)	0	9 (0.1)	11 (0.1)	3 (1-3) [4]	2.2 (1.5)
Neurology	17 (4.6)	9 (6.8)	126 (7.0)	72 (5.8)	18 (5.1)	242 (3.3)	467 (4.3)	19 (10-37) [82]	27.5 (22.6)
Obstetrics and gynecology	5 (1.3)	0	21 (1.2)	12 (1.0)	2 (0.6)	33 (0.5)	68 (0.6)	11 (9-22) [23]	13.4 (8.0)
Ophthalmology	21 (5.6)	1 (0.8)	103 (5.7)	37 (3.0)	5 (1.4)	262 (3.6)	408 (3.8)	11 (8-19) [131]	19.4 (26.9)
Orthopedics	29 (7.8)	4 (3.0)	131 (7.3)	37 (3.0)	26 (7.3)	184 (2.5)	382 (3.5)	9 (4-15) [80]	13.2 (15.9)
Pathology	33 (8.8)	23 (17.4)	89 (4.9)	80 (6.5)	11 (3.1)	265 (3.7)	468 (4.3)	12 (5-21) [50]	14.2 (11.6)
Radiology	12 (3.2)	1 (0.8)	25 (1.4)	14 (1.1)	3 (0.8)	2 (0.03)	45 (0.4)	3.5 (1-6) [10]	3.4 (2.9)
Toxicology	1 (0.3)	0	1 (0.1)	0	0	0	1 (0.01)	NA	NA

Abbreviation: NA, not applicable.

From 2008 to 2021, 10 776 supplements to these PMA devices were approved, with a median of 2.5 (IQR, 1.2-5.0) supplements per device approved annually. The most common supplement type was 30-day, at 7250 (67.3%), while PTS was least common, making up 132 (1.2%) of all supplements. Among the remaining supplement types, 1801 (16.7%) were 180-day, 1239 (11.5%) were real-time, and 354 (3.3%) were special.

The most common reason for supplement submission was process changes, accounting for 7293 (67.7%) of all supplements. These are meant to address manufacturing procedures (eg, sterilization, automating, and joining materials).^15^ Device design changes were next most common, with 1635 supplements (15.2%), followed by labeling changes, with 837 (7.8%) (Table 2).

**Table 2.** FDA Reason for Device Modification by Supplement Type

Reason	Supplement type, No. (%)					
	Panel track	180-d	Real-time	Special	30-d Notice or 135-d	All
Total No.	132 (100)	1801 (100)	1239 (100)	354 (100)	7250 (100)	10 776 (100)
Design or components	18 (13.6)	614 (34.1)	997 (80.5)	5 (1.4)	1 (0.01)	1635 (15.2)
Labeling	111 (84.1)	344 (19.1)	171 (13.8)	211 (59.6)	0	837 (7.8)
Processing	2 (1.5)	31 (1.7)	49 (4.0)	121 (34.2)	7090 (97.8)	7293 (67.7)
Postapproval study protocol	0	463 (25.7)	0	2 (0.6)	0	465 (4.3)
Change in manufacturing location	0	331 (18.4)	0	0	0	331 (3.1)
Other	1 (0.8)	18 (1.0)	22 (1.8)	15 (4.2)	159 (2.2)	215 (2.0)

Abbreviation: FDA, US Food and Drug Administration.

During the study period, 97 devices (26.0%) were recalled, with a median time until first recall of 1.9 (IQR, 1.0-2.9) years. These 97 devices caused 193 recalls, due to 66 (17.7%) devices undergoing multiple recalls, with 1 device undergoing 26 in the study period (Heartware ventricular assist system [Medtronic]). Cardiovascular devices accounted for 44 (45.4%) of the recalled devices, and 111 (57.5%) of all recalls.

Twenty devices (5.4%) experienced class 1 recall, of which 15 (75%) were cardiovascular devices. These 20 devices led to 43 class 1 recalls secondary to 6 (1.6%) having multiple class 1 recalls.

### Primary Outcome

**Figure 1.**
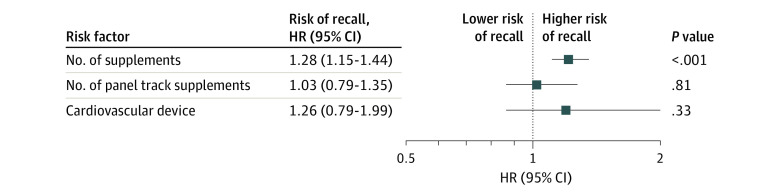
Multivariable Analysis of Risk Factors for All Recall Classes Hazard ratios (HRs) express the risk of recall associated with an increase of 1 supplement or panel track supplement per year and for cardiovascular devices, with other device type as the reference value.

Increased number of supplements, increased number of PTS, and classification as a cardiovascular device vs other type of device were all shown to be associated with increased risk of recall in univariable analysis, with hazard ratios (HRs) of 1.31 (95% CI, 1.18-1.45; *P* < .001) for each increase of 1 supplement per year, 1.94 (95% CI, 1.09-1.70; *P* = .005) for each increase of 1 PTS per year, and 1.34 (95% CI, 1.22-3.10; *P* = .005) for cardiovascular devices. Multivariable analysis, however, found supplement number as the only associated variable (HR, 1.28 [95% CI, 1.15-1.44]; *P* < .001) after accounting for cardiovascular device and PTS (Figure 1).

**Figure 2.**
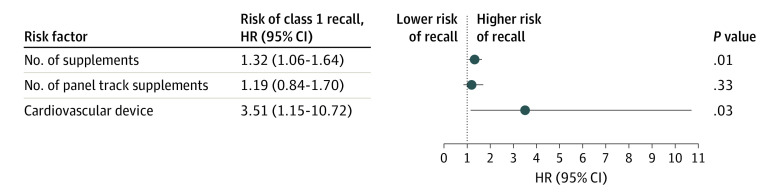
Multivariable Analysis of Risk Factors for Class 1 (High-risk) Recall Hazard ratios (HRs) express the risk of class 1 recall associated with an increase of 1 supplement or panel track supplement per year and for cardiovascular devices, with other device type as the reference value.

With respect to only class 1 recalls, supplement number, PTS number, and cardiovascular device supplements were again associated in univariable analysis, with HRs of 1.43 (95% CI, 1.18-1.74; *P* < .001) for each increase of 1 supplement per year, 1.56 (95% CI, 1.23-1.99; *P* < .001) for each increase of 1 PTS per year, and 6.11 (95% CI, 1.85-20.23; *P* = .003) for cardiovascular devices. Multivariable analysis, however, demonstrated both supplement number (HR, 1.32 [95% CI, 1.06-1.64]; *P* = .01) and cardiovascular device (HR, 3.51 [95% CI, 1.15-10.72]; *P* = .03) to be associated after accounting for panel track supplement in the model (Figure 2).

### Reasons for Recall

As a secondary aim, we investigated FDA-determined root causes for recall. There were 27 unique root causes identified in the study group. Device design was the largest percentage, accounting for 56 recalls (29.0%), and 23 (53.5%) of class 1 recalls. Process control and software design were the next most common causes, contributing to 22 (11.4%) and 21 (10.9%) of all recalls, respectively (Table 3).

**Table 3.** Top 5 FDA-Determined Root Causes of Recall^a^

FDA root cause	Total, No. (%)	
	Recalls	Class 1 recalls
Total No.	193 (100)	43
Device design	56 (29.0)	23 (53.5)
Process control	22 (11.4)	5 (11.6)
Software design	21 (10.9)	2 (4.7)
Nonconforming material or component	15 (7.8)	1 (2.3)
Under investigation by firm	13 (6.7)	3 (7.0)
Other	66 (34.2)	9 (20.9)

Abbreviation: FDA, US Food and Drug Administration.

^a^ A total of 27 root causes were identified. The top 5 are shown, with the remainder combined in the other category.

## Discussion

The primary findings of this analysis indicate that postapproval modifications to high-risk medical devices approved through PMA are associated with the risk of recall. An increase of 1 supplement per year, on average, was associated with a 28% increased risk of any recall, and a 32% increased risk of class 1 recall. While prior cases of supplements leading to recall have been reported, this is the first study, to our knowledge, demonstrating an independent association between postapproval modifications and increased risk of recall and high-risk recalls.^7,8,9^ An estimated 10% of US residents will have medical devices implanted during their lifetime, and physicians should be aware of this association because many devices undergo extensive postapproval modifications.^16^

The accrual of supplements over time has raised concerns that the modern device may bear little resemblance to its approved predecessor. We found a median of 2.5 supplements were approved per device per year, similar to previous reports in the literature.^7,8,9^ Both the Sprint Fidelis and Riata cardiac leads were approved through supplements (180-day and real-time, respectively) to devices originally approved in the mid-1990s that each had 80 or more supplements through 2012. These devices were linked to multiple patient deaths and were recalled in 2007 and 2011, respectively, after implantation in hundreds of thousands of patients worldwide.^7,17^ Similarly, a total knee replacement system (New Jersey LCS [DePuy]) underwent 135 postmarket changes before it was withdrawn from the market due to high revision rates.^7,8^

Two-thirds of supplements were 30-day notice, all of which were submitted for process changes, such as changes to sterilization techniques or annealing methods. This is higher than the 28% to 47% reported in previous literature, although this can be attributed to those analyses including dates prior to the creation of 30-day notice in 1997.^7,8,9^ Several devices were recalled during the study period for sterility concerns, including a vascular closure device (EXOSEAL [Cordis]) in 2013. Interestingly, seven 30-day supplements were passed prior to its recall, including supplement S007 for “alternate sterilization minimum dose.” Despite passing 6 weeks before the recall, the limited information available precludes defining an association. The next most common supplement types were 180-day, followed by real-time, contributing 16.7% and 11.5%, respectively. Interestingly, 997 of 1635 device changes (61.0%) were handled via real-time, while 614 (37.6%) were 180-day, coinciding with previously published literature.^7,8,9^ The reasons for these proportions are not well understood but would generally imply that most design changes are considered minor, since a majority are approved via the less stringent real-time pathway. This is worthy of further investigation, considering the most common cause of recall was found in our study to be device design.

Other authors have expressed concerns regarding the paucity of clinical evidence supporting these changes, with only PTS requiring clinical data.^18^ We found PTS constituted 1.2% of supplements, consistent with the 0.2% to 1.0% reported in the literature.^7,8,9^ Furthermore, Zheng et al^18^ recommended the quality of trials supporting PTS should be improved, with less than half being randomized, and only one-third being blinded. We did not find an association between PTS and the risk of recall or class 1 recall, implying that, despite reported weaknesses, the clinical data may provide some protective effect, considering PTS are higher-risk changes and receive greater scrutiny post-approval.

Several other important secondary findings are worth mentioning. First, the median time until first recall was less than 2 years from original device approval. This is consistent with findings from Somberg et al,^19^ who reported 71% of PMA device recalls occur within 3 years of original approval. This is concerning, considering the rapid integration of novel technologies into clinical use. Lampert et al^20^ found that two-thirds of implantable cardiac defibrillators were the most current model. An analysis of the National Health Service in England^21^ found that 45% of the marketed total hip arthroplasty brands were on the market for fewer than 3 years, and half of those lacked any clinical effectiveness data.

Second, cardiovascular devices had 3.5 times the risk of a class 1 recall. Zuckerman et al^22^ found that 31% of class 1 recalls were attributable to cardiovascular devices, although they included devices approved via any FDA pathway, including 510(k). This was the first study identifying cardiovascular devices as a risk factor for class 1 recall.^22^ Interestingly, we did not find cardiovascular devices to be associated with greater risk of recall when combing all classes of recall, likely reflecting the elevated risks that these devices possess in addressing cardiovascular conditions in typically high-risk patients compared with other, lower-acuity fields such as ophthalmology or orthopedics.

Third, device design flaws caused nearly 30% of recalls, and over one-half of class 1 recalls, making it the largest root cause. This appears consistent with several smaller studies in the orthopedic and radiological specialties.^23,24^ This likely underestimates the contribution of product design in device failures, though. We identified 27 root causes of recall assigned by the FDA, often with overlapping terms such as *device design* vs *component design* or *labeling design* vs *labeling error*. While the FDA publishes a brief manufacturer’s reason for recall, it usually added little clinical insight with which to analyze the recall from a clinician’s perspective. For instance, it is not readily apparent why process control was the root cause of recall for an embolization device (Pipeline [Micro Therapeutics Inc]) indicated for intracranial aneurysms when the manufacturer reports that the device “may fracture at the distal section during device implantation.”^25^ Several similar examples of seeming inconsistencies were noted during our analysis but are beyond the scope of our current investigation.

### Limitations

There are important limitations to this study. First, it is a retrospective, large-database, observational cohort analysis and cannot prove causation. Uncontrollable factors such as frequency of device use could influence both the number of supplements and recalls. By using a time-to-event analysis, we believe we provide the strongest evidence possible regarding the role supplements play in medical device recall. Next, available data from the FDA are limited, further challenging identification of a causal relationship between supplements and recall. For instance, the database reports a left ventricular assist system (Heartmate II [Thoratec]) was issued a class 1 recall in 2012 for a “trend of disconnected bend reliefs on the sealed outflow graft,” but does not indicate that it was a result of a 180-day supplement passed in 2010 described in the database as a “design change to the pump end bend relief region.”^26,27^ Most recalls, however, do not receive similar analysis in the published literature, making definitive determinations about an association impossible without more information from the FDA or the manufacturer regarding the precise reason the device failed.

The ability of recalls to accurately measure device safety has also been questioned.^2^ While used frequently in the literature, recalls likely underestimate the true nature of device-related complications. Nearly half of FDA Medical Device Safety Communications are identified from adverse event reports from MedWatch or MAUDE (Manufacturer and User Facility Device Experience), which the FDA cautions is a “passive surveillance system [that] has limitations, including the potential submission of incomplete, inaccurate, untimely, unverified, or biased data”.^28,29^ Further underestimation may occur when a safety concern does not meet the FDA’s risk threshold for recall. In these instances, the FDA can issue a safety communication or a Letter to Health Care Providers.^30^ Additionally, manufacturers can withdraw a device from the market for a “minor violation that would not be subject to legal action by the FDA.”^13^ There were 32 withdrawn devices found in this study, including an integrated dual balloon system used in weight loss surgery (ReShape [Warden Bariatrics]), which was withdrawn after the FDA published a Letter to Health Care Providers warning of reported cases of pancreatitis and death in patients receiving the device.^31^ To minimize subjectivity, we included only those devices formally recalled in our end point. Last, important regulatory changes occurred over the 13-year study period, although in the only study investigating risk of recall over time, Ghobadi et al^32^ found no significant increased risks of recall in the time periods between passage of the Medical Device User-Fee Amendments Act II (2007-2012) and Act III (2012-2016).

Our results should not be misconstrued to imply that the regulatory process is intrinsically flawed. The supplement process is critical in expediting delivery of incremental device changes with potentially greater safety and effectiveness profiles. Rather, we believe physicians should be aware of the risks associated with modified devices for their practices. In addition, we agree with recommendations to strengthen postmarket surveillance.^6,7^ Integrating the Unique Device Identifications that are now mandated on most modern implants into electronic medical records would facilitate tracking and identifying problematic devices.^6,33^ Improving registry participation can also facilitate failure detection, as seen with the metal-on-metal hips.^34^ The American Academy of Orthopedic Surgeons’ American Joint Replacement Registry is one of the largest in the world, but captures only an estimated 40% of arthroplasty surgical procedures in the US.^35^ Finally, both the FDA and manufacturers should provide higher-quality data regarding device recalls that include not just the reason for recall, but a synopsis of how and why the device or process failed.

## Conclusions

The findings of this cohort study suggest that postmarket modifications to high-risk medical devices may increase their risk of recall and class 1 recall. Physician awareness, improved preapproval testing, and postmarket surveillance strategies should be used to mitigate risks to patient safety and public health.

## Notes

Supplement.
Data Sharing Statement
