# Error in Author Affiliations

**DOI:** 10.1001/jamanetworkopen.2023.13606

**Published:** 2023-05-05

In the Original Investigation titled “Risk of Recall Associated With Modifications to High-risk Medical Devices Approved Through US Food and Drug Administration Supplements,”^1^ published April 12, 2023, Dr Enriquez’s author affiliations were incorrect. This article has been corrected.
