# Supplementary material for: Risk of Recall Associated With Modifications to High-risk Medical Devices Approved Through US Food and Drug Administration Supplements
Source: JAMA Netw Open. 2023 Apr 12;6(4):e237699. doi: 10.1001/jamanetworkopen.2023.7699 (PMC10099049; doi:10.1001/jamanetworkopen.2023.7699)
Supplement: Supplement. — Data Sharing Statement [file jamanetwopen-e237699-s001.pdf]

Data Sharing Statement

Dubin. Risk of Recall Associated With Modifications to High-Risk Medical Devices Approved Through US Food and Drug Administration Supplements. *JAMA Netw Open*. Published April 12, 2023. doi:10.1001/jamanetworkopen.2023.7699

Data
Data available: No
